# Supplementary material for: Unveiling the Effect of Solvents on Crystallization and Morphology of 2D Perovskite in Solvent-Assisted Method
Source: Molecules. 2022 Mar 11;27(6):1828. doi: 10.3390/molecules27061828 (PMC8951341; doi:10.3390/molecules27061828)
Supplement: Supplementary file 1 [file molecules-27-01828-s001.zip › molecules-1623405-supplementary.pdf]

Unveiling the Effect of Solvents on Crystallization and Morphology of 2D Perovskite in Solvent-Assisted Method

Yingjie Su [1], Jianqiang Xue [1], Anmin Liu [1], Tingli Ma [2,3] and Liguo Gao [1,*]

[1] State Key Laboratory of Fine Chemicals, Dalian University of Technology, Dalian 116023, China; suyingjie@mail.dlut.edu.cn (Y.S.); dut_jianqiang_xue@163.com (J.X.); anmin0127@163.com (A.L.)

[2] Department of Materials Science and Engineering, China Jiliang University, Hangzhou 310018, China; tinglima@dlut.edu.cn

[3] Graduate School of Life Science and Systems Engineering, Kyushu Institute of Technology, Kitakyushu 808-0196, Fukuoka, Japan

* Correspondence: liguo.gao@dlut.edu.cn

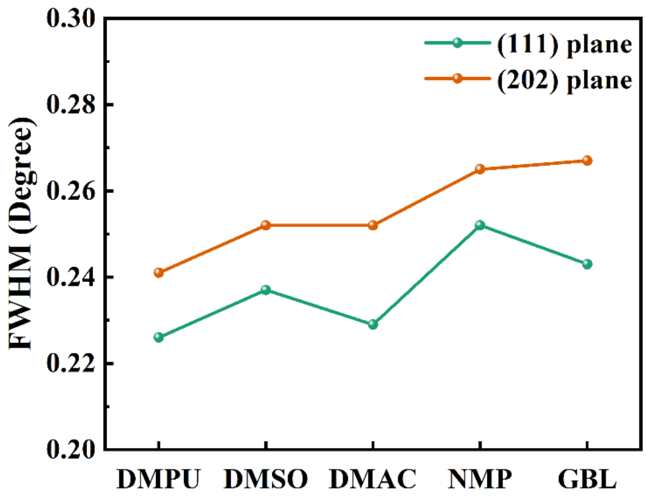

Figure S1. The FWHM of the (111) and (202) diffraction peaks in the XRD patterns. The crystal sizes of perovskite based on DMPU, DMSO, DMAC, NMP and GBL were 57.6 nm, 51.1 nm, 50.3 nm, 47.7 nm and 40.9 nm respectively, which were calculated according to the Scherrer formula.

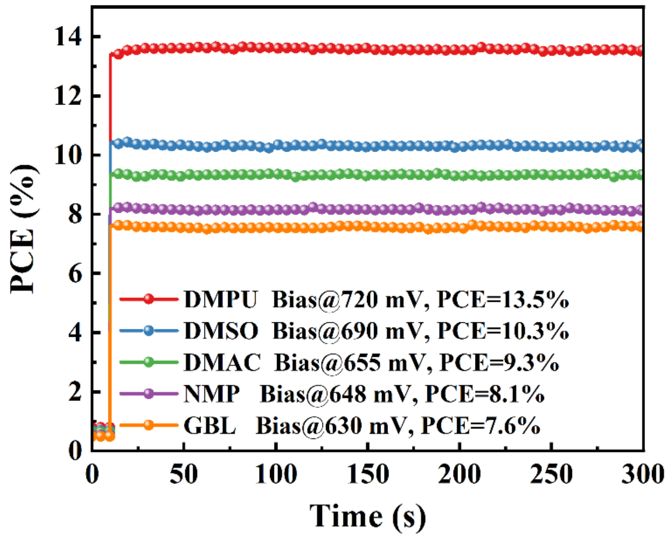

Figure S2. The stabilized power output of devices prepared by different solvents, under simulated AM 1.5G illumination of 100 mW/cm^2.

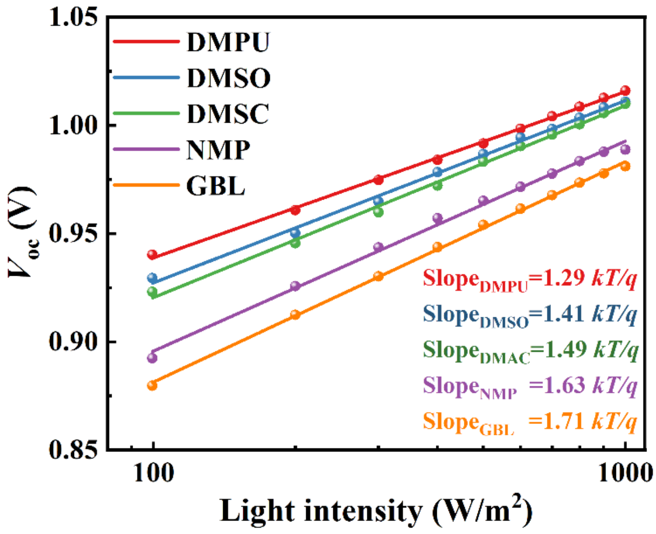

Figure S3. V_{oc} versus light intensity characteristics of devices prepared by different solvents.

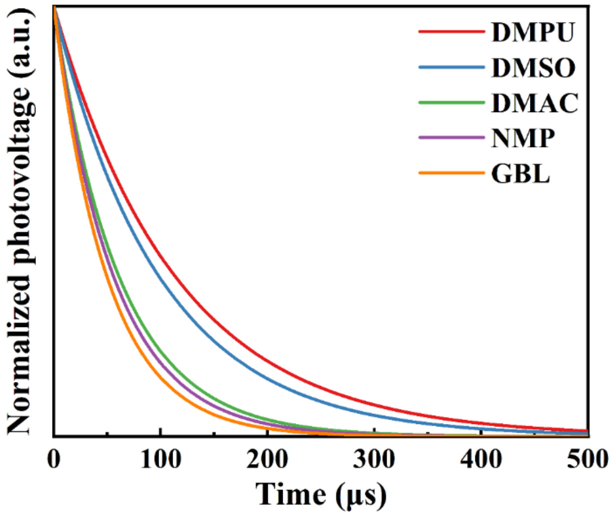

Figure S4. Normalized TPC curves of devices prepared by different solvents.

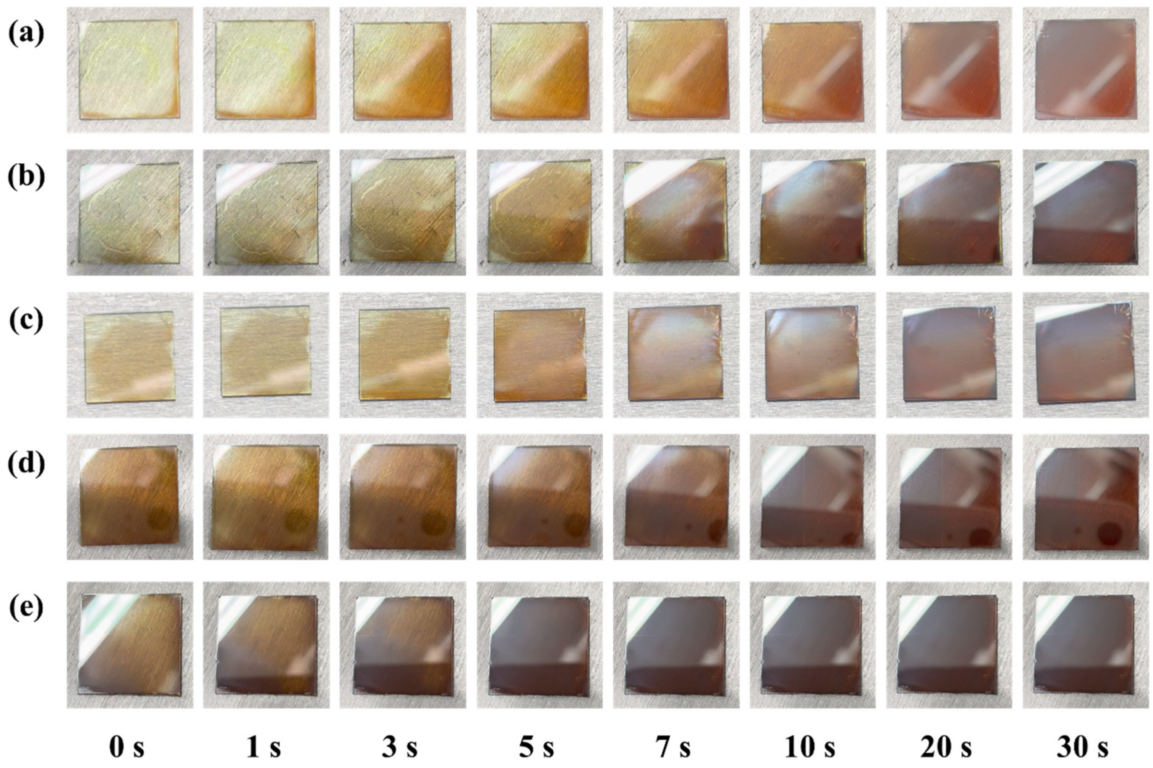

Figure S5. Optical images for perovskite crystalline growth with different time on 100°C hot plate, (a) DMPU; (b) DMSO; (c) DMAC; (d) NMP; (e) GBL.

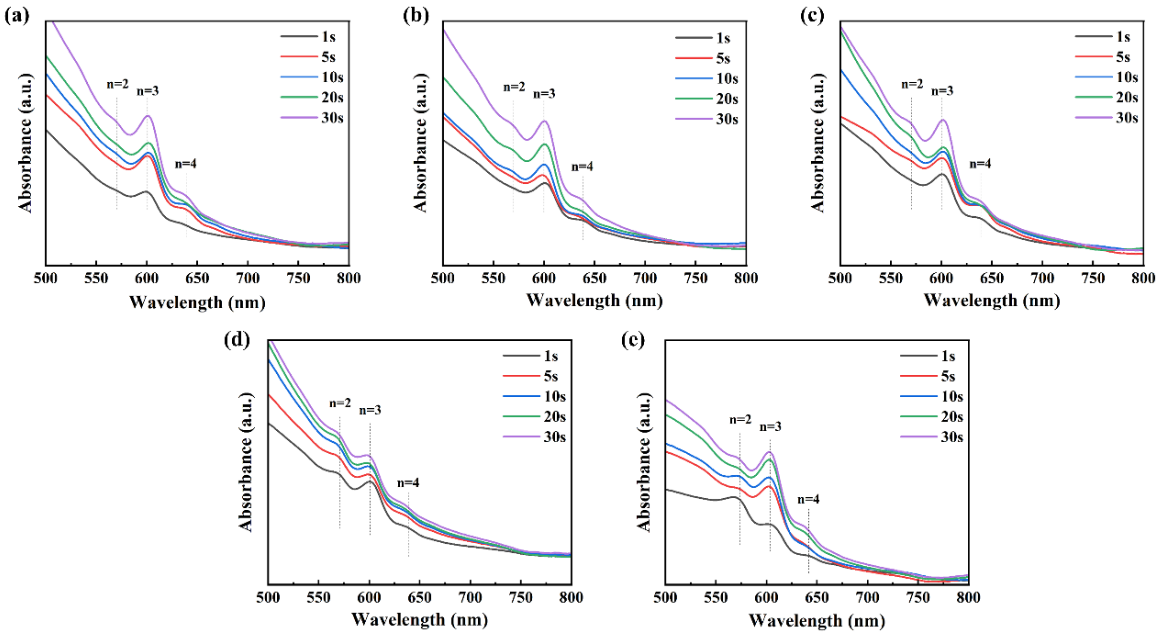

Figure S6. UV-vis absorption spectra of perovskite films with time variation, (a) DMPU; (b) DMSO; (c) DMAC; (d) NMP; (e) GBL.

Table S1. The molecular structural formula and properties of five assistant solvents. Among them, the dielectric constant and Gutmann's donor number represent the polarity and the Lewis basicity of solvents, respectively.

	**molecular structural formula**	**boiling point (℃)**	**dielectric constant**	**Gutmann's donor number (kcal/mol)**
**DMPU**	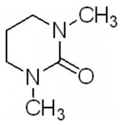	246	37.5	34.0
**DMSO**	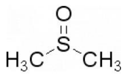	189	48.9	29.8
**DMAC**	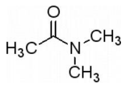	166	38.7	27.8
**NMP**	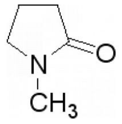	202	32.2	27.3
**GBL**	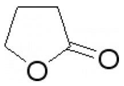	204	36.3	18.0

Table S2. Carrier recombination resistance (R_{rec}) and the trap state density (N_t) in devices based on different assistant solvents.

	**DMPU**	**DMSO**	**DMAC**	**NMP**	**GBL**
**R_{rec} [kΩ]**	~22.8	~10.6	~8.3	~4.0	~1.9
**N_t [10^{15} cm^{-3}]**	0.66	0.97	1.41	1.74	2.10

Table S3. Single exponential fitting parameters of TPC and TPV decays of devices prepared by different solvents.

	**DMPU**	**DMSO**	**DMAC**	**NMP**	**GBL**
**τ_{TPC} [μs]**	0.099	0.160	0.195	0.333	0.392
**τ_{TPV} [μs]**	114.82	99.56	61.69	56.60	50.28
